# Impact of a Strength Training Program on Physical Performance in U10 Soccer Players: A Quasi-Experimental Trial

**DOI:** 10.3390/children12091200

**Published:** 2025-09-08

**Authors:** Adrián Torregrosa-Domínguez, Iván Moreno-Camacho, Eduardo José Fernández-Ozcorta, Rafael Ramos-Véliz

**Affiliations:** 1 Facultad de Educación, Psicología y Ciencias del Deporte, Universidad de Huelva, Av. de las Fuerzas Armadas S/N, 21007 Huelva, Spain; adrian.torregrosa@alu.uhu.es (A.T.-D.); ivan.moreno846@alu.uhu.es (I.M.-C.); 2 Centro de Estudios Universitarios Cardenal Spínola, Fundación CEU San Pablo Andalucía, 41930 Bormujos, Spain; rramos@ceu.es; 3 Fundación San Pablo Andalucía, University CEU Fernando III, 41930 Bormujos, Spain

**Keywords:** academy soccer, grassroots, physical development, relative age effect, intervention, methodology

## Abstract

**Highlights:**

**What are the main findings?**

- A playful strength training program integrated into soccer practice produced greater gains in handgrip strength than usual training.
- Agility and lower-body power improved only within the intervention group, with no significant differences versus the controls.
- BMI decreased in the intervention group, but between-group differences were not significant due to higher baseline BMI.
- RAE did not influence training response in this sample.

**What is the implication of the main finding?**

- Early strength training in grassroots soccer is safe, feasible, and effective for handgrip strength.
- Coaching curricula should include strength development early, while further research is needed on transfer to agility, jump performance, and BMI.

**Abstract:**

**Background/Objectives:** The integration of strength training in grassroots youth soccer remains limited, often due to persistent myths regarding its safety and utility. This study evaluated the effectiveness of a tailored, playful strength training program in young players and analyzed the influence of the Relative Age Effect (RAE) on physical development and training response. **Methods:** A 14-week quasi-experimental pretest–posttest design was conducted with 27 federated male soccer players aged 9–10 years (experimental: *n* = 15; control: *n* = 12). The intervention consisted of twice-weekly, game-based strength training sessions integrated into the regular team routine. Outcomes included validated anthropometric (BMI) and functional (handgrip strength, standing long jump, Illinois agility test) indicators. RAE was analyzed according to birth quartile. Non-parametric statistical analyses and effect size (r) calculations were used. **Results:** The experimental group achieved significantly greater pre–post gains in handgrip strength than controls (right and left). Agility and standing long jump improved within the experimental group, but between-group differences were not significant. BMI decreased within the experimental group, yet the net between-group difference in BMI change was not significant in the context of a higher baseline BMI (*p* = 0.047). Although the Relative Age Effect (RAE) influenced baseline BMI, no moderating effect was detected on performance variables or training-induced changes in this sample. **Conclusions:** In U10 soccer players, a playful, context-integrated strength program produced superior gains in handgrip strength compared with usual practice. Improvements in agility and standing long jump were observed within the intervention group, but did not exceed those of the controls. BMI changes were not different between the groups and must be interpreted with caution, given baseline imbalances. In this sample, RAE did not moderate training response.

## 1. Introduction

Although soccer has established itself as a global social phenomenon, discussions on physical preparation at the youth level remain anchored in traditional paradigms. The historical primacy of technical and tactical training in soccer academies contrasts with a growing body of evidence that highlights the importance of strength, power, and agility as essential conditions for both athletic success and health in the contemporary era [1,2,3]. Recent literature emphasizes that modern soccer is inherently intermittent and explosive, requiring not only technical skills but also high levels of physical fitness [4].

It is paradoxical that, at the foundational levels of this sport, the notion still prevails that physical conditioning—especially strength training—is unnecessary or even potentially harmful in early ages. This perspective, often justified by presumed risks to growth or by the supposed lack of competitive relevance of strength in childhood, has been widely refuted in the specialized literature [5,6]. Nevertheless, cultural resistance persists, hindering the implementation of evidence-based practices. Recent systematic reviews confirm that well-designed strength programs in children are both safe and effective, supporting not only physical and motor improvements but also cognitive and psychosocial benefits [7]. This persistence may suggest that opposition to strength training is rooted more in institutional inertia and unfounded fears than in robust scientific arguments.

The epidemiological context adds another layer of complexity: sedentarism and the arrival of children to clubs with motor and strength deficits are increasing problems in many regions [8]. This reality affects both motor development and overall health, as well as long-term engagement in sports participation [9]. Furthermore, approaches such as the Long-Term Athletic Development (LTAD) model advocate for shifting the focus away from technical repetition towards a holistic perspective, where motor competence and strength training are central [6]. However, this conceptual transition still faces resistance in the day-to-day practice of many clubs.

It should also be noted that the controversy is not about whether children can or should engage in strength training, but rather about how, when, and under what conditions it is most appropriate and safe to do so [10,11]. There is consensus that interventions should be playful, individualized, supervised, and contextualized within the grassroots soccer ecosystem. Nonetheless, the literature remains ambiguous regarding the optimal dose, best methodological sequence, and the actual degree of transfer from strength programs to relevant functional indicators.

These debates are further complicated by the issue of equity in talent development, particularly regarding the “Relative Age Effect” (RAE). Although RAE is widely recognized as a potential source of inequality in the identification and promotion of young soccer players, its influence on the response to physical intervention programs remains underexplored, and when addressed, results tend to be inconsistent [12,13].

This study is based on the hypothesis that a strength training program, adapted and contextualized within grassroots soccer, can produce measurable improvements in strength, agility, and anthropometric markers. Furthermore, such interventions may help to reduce traditional barriers to talent promotion, provided they are evaluated using criteria of equity and empirical evidence. In particular, we critically examine the real impact of RAE as a modulating factor in outcomes, challenging the widespread belief that it decisively shapes physical development and performance in children.

The originality of the present work lies in situating empirical analysis within a real and uncontrolled club context, adopting a critical perspective on the limits of transferring theory into practice. The aim is not to offer a closed model of intervention, but to challenge existing assumptions and contribute evidence for both practical decision-making in clubs and the academic debate on equity and physical development in youth soccer.

This manuscript focuses on the evaluation of a strength training program in 9- to 10-year-old male federated players in a Spanish grassroots soccer club over a 14-week period, with handgrip strength, lower-body power, agility, and BMI as main variables.

## 2. Materials and Methods

### 2.1. Study Design

A quasi-experimental pretest–posttest study with non-equivalent groups over a period of 14 weeks was conducted. This approach, while not a pure randomized experimental design, was necessary due to the real-world structure of grassroots soccer: teams were already formed within their sports academies, making random assignment impossible. This type of design brings inherent limitations—mainly the risk of selection bias and reduced internal validity—but it preserves contextual authenticity and real-world applicability of the findings. In applied sports science, strict experimental rigor must often be balanced with feasibility and practical impact, as recommended by quality guidelines for field research.

The experimental group participated in a strength training intervention integrated into their weekly team routine, while the control group continued their usual training and served as a natural reference for typical development. Both groups were assessed at three time points: pretest (week 0), mid-intervention (week 7), and posttest (week 14), allowing us to track change trajectories rather than just point differences.

The study complied with the principles of the Declaration of Helsinki to protect data and participants’ rights. The protocol was reviewed and approved by the Andalusian Bioethics Committee (SICEIA-2024-001206). All legal guardians gave written informed consent after receiving clear and accessible information, in line with international recommendations for research with vulnerable populations.

### 2.2. Participants and Sample

The study included male youth soccer players aged 9 to 10 years (Mean = 8.91 ± 0.44). The total sample consisted of 27 players, who were not randomly divided into two groups, as they were already participating in a specific team: the experimental group (*n* = 15; mean age 9.02 ± 0.56 years) and the control group (*n* = 12; mean age 8.78 ± 0.25 years).

All participants had at least one year of experience playing federated Soccer. The selected teams trained three times per week (Tuesday, Thursday, and Friday, 17:15–18:30), in addition to playing an official competitive match every weekend. All players need to attend training regularly to be included in the study. No major adverse events occurred.

Recruitment was conducted through the club’s coaches and coordinators, who informed the players and their families about the study’s objectives and conditions.

### 2.3. Variables and Instruments

The selection of tests and variables was based on criteria of validity, clinical relevance, and feasibility in real-world settings, following the recommendations of the ALPHA-Fitness battery and international literature [14]:

Anthropometric markers. Weight and height were measured using calibrated instruments, and BMI was calculated using the standard formula (kg/m^2^). While BMI is widely used as an epidemiological marker, it has limitations for detecting functional changes in this age group and should be interpreted with caution [15]. However, BMI was used as it has been recommended for this age group in the ALPHA-Fitness battery and international literature [14]. Other strategies were not possible due to the children’s schedules, as the tests are typically conducted in the morning. Additionally, we had a lack of resources for kinanthropometric measures. Above all, we prioritized the practicality of BMI, which can be readily assessed by coaches without specialized knowledge.

Handgrip strength (dominant and non-dominant). A hand dynamometer was used, with two attempts per hand, and the mean value recorded. This test, widely validated in children, is sensitive to motivational factors and motor learning [16].

Standing long jump. An indicator of lower-body power, measured as the mean of two attempts. This test detects relevant functional improvements and is minimally invasive.

Agility (Illinois Agility Test). Assesses the ability to change direction and speed in a game-like context, using two timed attempts and calculating the average [17].

In addition, the Relative Age Effect (RAE) was calculated by assigning participants to birth quartiles according to the academic calendar, allowing for analysis of the potential impact of relative age on performance and training response.

### 2.4. Procedure

The evaluation process was organized into several phases to ensure not only the quality and reliability of the collected data but also strict adherence to ethical standards and the logistical realities of youth sports settings. As an essential first step, informed consent was obtained from the parents or legal guardians of all participants; no child was included without this authorization, in line with ethical regulations for research involving minors.

Once documentation was complete, three key assessment sessions were scheduled: one before the intervention (pretest), one 7 weeks mid-intervention (re-test), and one immediately after its completion (posttest), with efforts made to replicate initial conditions in both sessions. Whenever possible, measurements were conducted at the same time and place to minimize environmental variations that could bias results.

Before starting the battery of tests, anthropometric data (weight and height) were collected in a reasonably private setting, with participants wearing only underwear and no shoes. All instruments (scales, stadiometer, hand dynamometer) were calibrated according to the manufacturer’s instructions at the beginning of each assessment day. Any detected discrepancies were addressed prior to data collection to ensure measurement accuracy.

The process was supervised by two experienced evaluators in pediatric sports assessment, who ensured consistency in instructions and test administration. Both received specific training before the study and carried out joint simulations to unify criteria and resolve potential procedural doubts. Familiarization sessions were conducted a few days before formal testing, so that the children already understood the procedures and instructions, likely reducing anxiety and resulting in more consistent outcomes.

After weighing and measuring, all participants completed a brief structured warm-up: five minutes of joint mobility and dynamic games, followed by two to three minutes of general activation exercises, to facilitate a smooth transition to the more demanding tests. To streamline data collection without disrupting regular training, participants were divided into three subgroups rotating through different stations: Illinois Agility Test, standing long jump, and handgrip strength test (right and left hand, always using the same dynamometer and supervision). This rotation system allowed all children to complete the three main tests within about 20 min, avoiding long waits and reducing the risk of accumulated fatigue between tests.

Finally, data management was confidential and anonymized: each participant’s results were coded using an alphanumeric identifier accessible only to the lead investigators. Paper records were kept locked, and digital files were stored on password-protected servers. At the end of the process, all data were reviewed and cleaned to verify consistency and detect potential outliers before statistical analysis.

### 2.5. Intervention Program

Selecting an appropriate methodology for strength training program in grassroots soccer is far from trivial. The available evidence suggests that circuit-based programs, performed twice a week and well integrated into regular training sessions, can produce significant improvements in both explosive strength and muscular endurance [18]. Even in school settings, this type of intervention not only enhances motor engagement but also helps optimize the often limited time available at these ages [19].

In this study, a structured intervention (Table 1) was implemented over 14 consecutive weeks. This included two weekly strength training program sessions, always integrated into the team’s regular dynamics and framed within a playful, soccer-relevant approach. Following the rationale of Radnor et al. [6], all tasks prioritized the development of athletic motor skill competencies (AMSC) through game-based activities, problem-solving, and the use of bodily analogies (e.g., animal shapes, obstacles, cooperative and competitive challenges), deliberately avoiding overly rigid or artificial approaches that might diverge from the realities of youth training.

During the first four weeks, the focus was on familiarization and technical mastery of basic movement patterns. Each session (twice per week, 30–40 min) included 6–8 exercise stations, alternating between locomotion, reaction games, and general strength work using body weight (squats, assisted push-ups, lunges, planks), inspired by the logic of animal shapes and cooperative circuits. The volume was approximately 2–3 sets of 10–15 repetitions per exercise, with low intensities (RPE 3–4/10) and generous rest periods (at least 45–60 s) to ensure quality of execution and motor learning.

Between weeks five and ten, the workload was gradually increased. Slightly more demanding variants were introduced (jump squats, incline push-ups, single-leg bridges), along with specific agility tasks and soccer-contextualized change-of-direction exercises (reaction runs, relays, small-sided duels). The volume increased to 3–4 sets of 8–12 repetitions, intensity was raised to RPE 5–6/10, with 45 s of rest between stations and up to 90 s between blocks. This phase also alternated days dedicated to plyometrics (horizontal jumps, multi-jumps) and accelerations, so that each week children experienced both types of stimuli in a complementary manner.

The final four weeks focused on transfer and motor integration in contexts as similar as possible to real gameplay. Stations and circuits became more dynamic, including cooperation-opposition challenges (e.g., mini-games with the ball, rescue races, decision-making sprints) and tasks requiring the application of strength, agility, or power in open and variable situations. The volume was kept at 3 sets per station, with 8–10 higher-demand repetitions, and a target intensity of RPE 6–7/10. Rest periods were intentionally shortened (30–45 s) to simulate the intermittent fatigue of soccer and promote adaptation to effort.

Throughout the program, individual responses were monitored, with progressive adjustments to difficulty and technical correction always prioritized before increasing load or reducing rest. The alternation of tasks, playful organization, and constant reference to the game aimed not only to promote physical adaptation but also to maintain children’s motivation and engagement—factors identified in the literature as critical for the success of such interventions [6,19,20].

### 2.6. Statistical Analysis

Data analysis was performed using Jamovi (Version 2.4.8), a user-friendly, open-source platform for statistical computing. All graphical representations were created with R Studio (Version 1.4.1717), employing the packages stats (Version 4.4.3), pwr (Version 1.3.0), and ggplot2 (Version 3.5.0).

The normality of continuous variables was examined using the Shapiro–Wilk test. Due to the small sample size and frequent deviations from normality, all main analyses were conducted using non-parametric tests. The Wilcoxon signed-rank test was used to compare within-group changes across time points (pre–mid, mid–post, pre–post), while the Mann–Whitney U test was applied for between-group comparisons of change (i.e., the net “difference in differences” for pre–post change). For the effect of relative age quartile (RAE), the Kruskal–Wallis test was used.

All outcome data are presented as median and interquartile range (IQR) for each time point (pre, mid, post). Within each group, absolute changes (Δ) for pre–mid, mid–post, and pre–post were calculated and reported, as well as the percentage change for the pre–post interval. The net difference in change (ΔΔ) between groups was computed for each outcome to quantify the effect attributable to the intervention.

Effect sizes for non-parametric tests were reported as the *r* statistic (*r* = Z/√N). Interpretation of effect sizes follows conventional thresholds, where *r* values of 0.1, 0.3, and 0.5 are considered small, medium, and large effects, respectively. However, given the small sample and clustered data, both absolute and percent changes in median were also reported for clinical relevance. The statistical significance level was set at *p* < 0.05 for all tests.

No statistical processes were needed to impute any missing data.

## 3. Results

### 3.1. Descriptive Analyzes

The study included a total of 27 participants, divided into an experimental group (*n* = 15) and a control group (*n* = 12). The median age in the experimental group was 9.0 years (IQR: 8.7–9.3), while in the control group it was 8.8 years (IQR: 8.6–9.0). Regarding initial anthropometric characteristics, the experimental group had a median stature of 135.0 cm (IQR: 129.5–139.0) and the control group 139.5 cm (IQR: 134.3–140.0). Median weight was 31.0 kg (IQR: 27.5–36.4) for the experimental group and 28.4 kg (IQR: 27.8–30.0) for the control group. The median BMI was 17.5 kg/m^2^ (IQR: 16.2–19.3) for the experimental group and 15.2 kg/m^2^ (IQR: 14.6–16.3) for the control group (see Table 2).

There was a statistically significant difference in BMI at baseline between groups (*U =* 49.0, *p* = 0.047), with the experimental group presenting a higher median BMI than the control group. No statistically significant differences were observed for right handgrip strength (*U =* 76.5, *p* = 0.526), left handgrip strength (*U =* 85.0, *p* = 0.826), agility (*U =* 65.5, *p* = 0.236), stature (*U =* 63.0, *p* = 0.193), or weight (*U =* 70.5, *p* = 0.354). Long jump showed a marginal difference (*U =* 49.5, *p* = 0.051), with slightly higher values in the control group (Median = 147.25 cm, IQR: 139.25–162.50) compared to the experimental group (Median = 131.50 cm, IQR: 125.50–145.25).

### 3.2. Primary Outcome Analysis

The results of the intervention are summarized below, with median and IQR values reported for each group. Statistical comparisons within and between groups are also presented, highlighting the main effects observed in the primary outcome variables (see Table 3).

Regarding changes in BMI, the experimental group started the intervention with a significantly higher median BMI than the control group (17.51 [IQR: 16.23–19.34] vs. 15.20 [14.63–16.25], *p* = 0.048). Both groups showed a reduction in BMI post-intervention: the experimental group decreased by Δ = −1.59 units (−9%, *p* < 0.05, Wilcoxon test), while the control group changed minimally and non-significantly (Δ = +0.03 units, +0.2%). Despite these reductions, the net between-group difference in change was ΔΔ = −1.29 units, which was not statistically significant (*U =* 62.0, *p* = 0.183, *r* = 0.31).

For right handgrip strength, the experimental group improved from 12.25 kg (IQR: 11.65–13.95) at pretest to 13.30 kg (IQR: 12.30–15.40) at mid-intervention (Δ Pre–Mid = +1.05), and further to 16.55 kg (IQR: 15.00–17.73) at posttest (Δ Mid–Post = +3.25; overall Δ Pre–Post = +4.30, +35.1%; *W =* 21.0, *p* < 0.001, *r* = 0.89). The control group increased from 12.18 kg (IQR: 9.88–13.96) to 13.35 kg (IQR: 11.01–16.06) at mid (Δ = +1.03), and to 13.56 kg (IQR: 11.25–16.54) at posttest (Δ = +0.30; overall Δ = +1.39, +11.4%; *W =* 18.0, *p* = 0.110, *r* = 0.54). The net between-group difference in change was statistically significant in favor of the experimental group (ΔΔ = +2.91; *U =* 37.5, *p* = 0.011, *r* = 0.58).

Similarly, the experimental group improved in left handgrip strength from 12.20 kg (IQR: 10.70–13.10) at pretest to 12.50 kg (IQR: 11.25–14.50) at mid-intervention (Δ = +0.30), and to 15.80 kg (IQR: 14.93–17.03) at posttest (Δ = +2.72; overall Δ = +3.60, +29.5%; *W =* 28.0, *p* < 0.001, *r* = 0.85). The control group shifted only slightly from 12.00 kg (IQR: 9.20–15.11) to 12.55 kg (IQR: 10.19–14.03) at mid (Δ = +0.05), and to 12.68 kg (IQR: 10.62–14.52) at posttest (Δ = +0.63; overall Δ = +0.68, +5.6%; *W =* 26.0, *p* = 0.339, *r* = 0.33). The difference in change between groups was highly significant (ΔΔ = +2.92; *U =* 28.0, *p* = 0.003, *r* = 0.69).

Both groups improved significantly within-group in Illinois agility test times, but the between-group difference was not statistically significant (ΔΔ = −0.59; *U =* 76.0, *p* = 0.510, *r* = 0.16).

Finally, regarding the standing long jump, the experimental group increased by +11.5 cm (Δ = +11.5 cm, +8.7%), while the control group showed only a minimal change (+0.5 cm, +0.3%). The between-group difference favored the experimental group numerically (ΔΔ = +11.0 cm) but was not statistically significant (*U =* 71.5, *p* = 0.379, *r* = 0.21).

**Figure 1:**
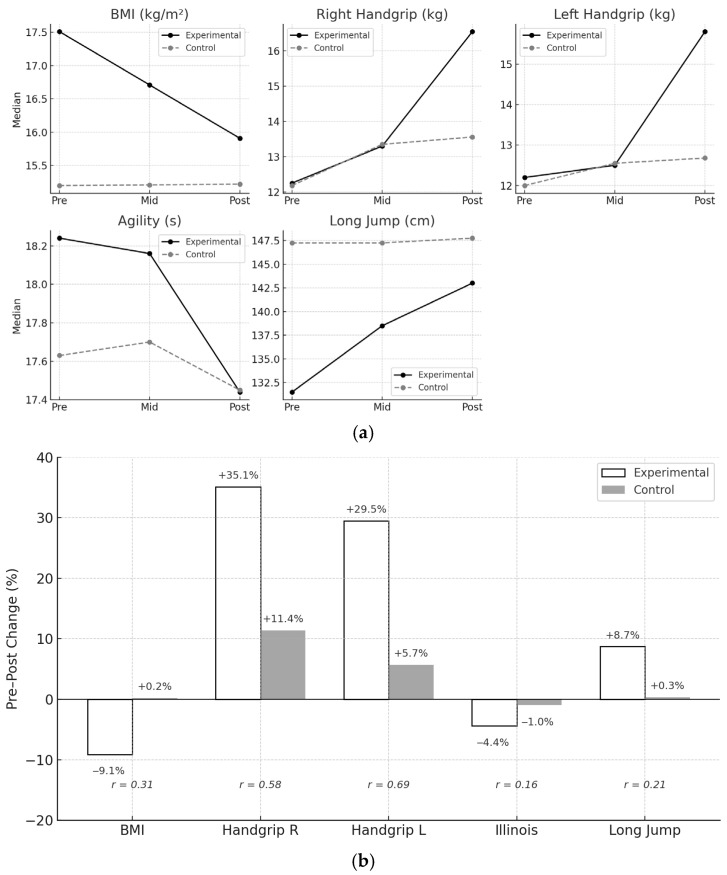
(**a**) Median values for functional outcomes at pretest, mid-intervention, and posttest for experimental and control groups; (**b**) Percentage pre–post change and between-group effect size (*r*) for each outcome. Numerical values above each bar indicate the median percentage change for each group; italicized values represent the effect size for the net difference (Mann–Whitney U, pre–post). Dashed lines indicate reference thresholds (−20% to +40%). Abbreviations: BMI = Body Mass Index; Right/Left Handgrip = handgrip strength; Illinois = Illinois Agility Test (lower values denote better performance); Long Jump = standing long jump.

Figure 1 provides both the temporal evolution and the magnitude of changes induced by the intervention, a composite figure was constructed. Panel A displays the median trajectories for each outcome at pretest, mid-intervention, and posttest, facilitating the visualization of longitudinal trends. Panel B summarizes the net pre–post change for each group and outcome, including the effect size (*r*) for between-group differences, thus enhancing interpretability for both clinical and research audiences.

### 3.3. Influence of Birth Quartile

In the experimental group, the distribution by birth quartile was: Q1 (*n* = 4, 26.7%), Q2 (*n* = 3, 20.0%), Q3 (*n* = 3, 20.0%), and Q4 (*n* = 5, 33.3%). In the control group, the distribution was: Q1 (*n* = 3, 25.0%), Q2 (*n* = 4, 33.3%), Q3 (*n* = 3, 25.0%), and Q4 (*n* = 2, 16.7%). The overall sample showed an even distribution by quartile (Q1: 25.9%, Q2: 25.9%, Q3: 22.2%, Q4: 25.9%). There were no significant differences in the distribution of birth quartiles between groups (χ^2^ = 1.25, *p* = 0.74).

The potential influence of birth quartile (Q1 to Q4) on both initial physical performance and observed changes was examined. Kruskal–Wallis tests revealed a significant difference in baseline BMI between birth quartiles (*p* = 0.002), while no significant differences were found for right handgrip strength (*p* = 0.297), left handgrip strength (*p* = 0.195), agility (*p* = 0.912), or long jump (*p* = 0.632). To assess whether birth quartile affected the pre–post change in BMI, it was included as a grouping factor in the Kruskal–Wallis test for change scores; no significant effect was found (Δ BMI, *p* = 0.879). Similarly, no significant birth quartile effect was observed for changes in right handgrip (*p* = 0.640), left handgrip (*p* = 0.556), agility (*p* = 0.833), or long jump (*p* = 0.637). Collectively, these results indicate that, although baseline BMI differed by birth quartile, RAE did not account for the observed improvements in physical performance.

## 4. Discussion

The fundamental aim of this study was to determine whether the integration of a tailored strength training program—specifically designed for children aged 8 to 10 and contextualized within grassroots soccer—could produce meaningful improvements in key physical capacities, including BMI, handgrip strength, agility, and lower limb power [6,11].

Our data indicate that the program clearly outperformed usual practice in handgrip strength, whereas agility and lower-limb power showed within-group improvements that did not translate into significant between-group differences. This pattern suggests specific efficacy for handgrip strength and limited or measurement-dependent transfer to agility and horizontal jump over 14 weeks, relative to an active control exposed to routine soccer practice.

The significant improvements observed in both dominant and non-dominant handgrip strength in the experimental group (*r* = 0.88 for both) exceed what would be expected from natural maturation or regular training, and are consistent with the effects reported by Radnor et al. [6] and Ben Othman et al. [20] in prepubescent participants undergoing structured interventions. Nevertheless, the magnitude of the effect warrants consideration of the possible contribution of test learning and participant motivation—phenomena well documented in pediatric contexts [16]. Indeed, part of the literature suggests that a significant proportion of early improvement in strength tests can be attributed to familiarization effects rather than genuine neuromuscular adaptations [16,21]. Therefore, while the data support the efficacy of the program, it is likely that a portion of the observed gains reflect a combination of specific adaptation and motor learning, rather than deep structural change.

Two non-exclusive explanations are plausible for the lack of between-group differences in agility and lower-limb power: (i) specificity and dose—a general, playful strength program may be insufficiently specific to outperform soccer practice in complex change-of-direction tasks or horizontal power within 14 weeks; (ii) measurement sensitivity and learning effects, as Illinois and long jump performance depend on technical execution and familiarization. Future studies should consider task-specific progressions, technique standardization, and a priori power for between-group contrasts.

Standing long jump improved only in the experimental group, in line with meta-analyses showing positive transfer of strength work to global athletic gestures in school-aged children [11,22]. However, the debate remains open regarding the minimum duration required to elicit structural adaptations versus improvements in motor efficiency alone [21,23]. Some studies highlight that, in short- or medium-duration programs, improvement is usually mediated by motor control rather than hypertrophy or deep neural potentiation [21]. In this sense, the 14-week duration used here may lie at the threshold where both mechanisms—motor learning and physical adaptation—overlap.

The experimental group recorded a statistically significant reduction in Illinois agility test times (Δ = −0.80 s, −4.4%), while the control group also improved significantly (Δ = −0.21 s, −1.2%). However, the between-group difference in change was not statistically significant (ΔΔ = −0.59; *U =* 76.0, *p* = 0.510). These results suggest that agility is trainable at early ages, provided that tasks are contextualized and varied [24,25]. Nevertheless, the literature notes that the transfer of training to standardized tests critically depends on the degree of similarity between the training context and the test itself [26]. It is plausible that the playful and functional design of the program facilitated both motor improvement and competitive motivation—factors that are difficult to isolate in field research [19].

The experimental group experienced a statistically significant reduction in BMI (Δ = −1.59, −9.1%), while the control group showed only a minimal change (Δ = +0.03, +0.2%). The net difference in change between groups was not significant (*U =* 62.0, *p* = 0.183, *r* = 0.31). It is noteworthy that the experimental group started with a higher median BMI (*p* = 0.048), which may have provided a greater potential for within-group reduction. This baseline imbalance is a threat to internal validity in a quasi-experimental design and should be acknowledged explicitly as a potential confounder. BMI should therefore be interpreted with caution, as it is a limited functional marker in childhood, susceptible to growth, diet, and sample size effects. However, this finding should be interpreted cautiously, as it aligns with literature warning about the limited sensitivity of BMI as a functional marker in childhood [15,27]. BMI can fluctuate due to factors unrelated to training, such as linear growth or dietary variations, and is particularly vulnerable to statistical distortions in small samples [27,28]. Therefore, it is likely that contextual factors—such as increased family awareness or changes in daily habits during the intervention period—contributed to the parallel tendency observed in both groups. This baseline imbalance is a threat to internal validity in a quasi-experimental design and should be acknowledged explicitly as a potential confounder.

A novel aspect of this study is the critical exploration of the RAE. Here, RAE affected baseline BMI but did not influence the other outcomes or the magnitude of improvement in any variable, partially consistent with the study’s findings that limited RAE impact in non-selective developmental stages or small groups [12,13]. However, the absence of effect should be interpreted with caution, as its influence may be greater in competitive selection settings [13]. Additionally, the small sample size could have masked subtle differences. It is also important to note that recent evidence indicates that maturational age, rather than chronological age alone, plays a decisive role in modulating physical and physiological responses to soccer training tasks in youth players, reinforcing the need for individualized training approaches according to biological development [29].

This study has several limitations that should be acknowledged. The small sample size and lack of randomization are primary constraints that limit internal validity and the generalizability of the findings [19,28]. This lack of randomization is particularly relevant when considering the baseline differences observed between groups, most notably the statistically significant disparity in BMI. It is plausible that the higher initial BMI in the experimental group indicated a lower baseline fitness level, thus offering a greater potential for improvement. However, this concern is mitigated by our primary analysis, which focused on the net difference in change (ΔΔ) between groups—a statistical approach that inherently controls for baseline disparities. Furthermore, significant gains were observed in variables less directly influenced by body mass, such as handgrip strength. Therefore, while the baseline BMI difference is acknowledged, the results suggest the observed improvements were predominantly an effect of the training program.

Beyond this specific imbalance, other uncontrolled external variables—such as diet, sleep, overall physical activity, or differential motivation and the Hawthorne effect—introduce uncertainty regarding the true magnitude of the effects attributable to the program [15,19]. To address these issues, future studies should employ randomized designs and incorporate multivariate adjustment for baseline values and other potential confounders, such as maturational status. Finally, given the limited sensitivity of BMI in childhood, subsequent research would benefit from including additional markers, like waist-to-height ratio, to characterize the effects of training interventions more accurately.

Despite its limitations, this work provides compelling arguments for reconsidering the marginal role of strength training in grassroots soccer. While the evidence supports the safety and effectiveness of well-designed programs [6,10], open questions remain regarding the minimum effective dose, sustainability of effects, and the interplay with educational and motivational factors [5]. These findings are further supported by recent systematic reviews, such as León-Reyes et al. [7], which confirm that strength interventions in children can yield broad benefits beyond athletic performance, including improved health, psychosocial well-being, and higher adherence to physical activity. However, the real-world implementation of such programs still requires careful adaptation to context, family engagement, and coach education—elements often overlooked in the controlled environments of published trials.

## 5. Conclusions

Taken together, the findings support superior effects on handgrip strength of a playful, integrated strength program in U10 soccer. Improvements in agility and standing long jump were observed within the intervention group, but were not superior to those of controls. BMI changes did not differ significantly between groups and must be interpreted with caution, given baseline imbalances. In this sample, RAE did not moderate training responses. Future randomized, adequately powered trials—with task-specific progressions and tighter control of confounders—are needed to clarify transfer to multidimensional performance.

## Figures and Tables

**Table 1:** Structure of the Strength Training Intervention Program by Phase.

Phase	Weeks	Main Objective	Exercises/Content	Sets × Reps	Intensity (RPE)	Rest Between Stations	Rest Between Blocks	Notes
Initial	1–4	Technical learning, familiarization, motor control	Squats, lunges, assisted push-ups, planks, locomotion, animal shapes, reaction, and chasing games	2–3 × 10–15	3–4/10	45–60 s	90 s	Playful and cooperative circuits, very gradual progression
Intermediate	5–10	Strength and agility progression, soccer integration	Jump squats, incline push-ups, single-leg bridges, relays, change in direction, reaction runs, plyometrics, accelerations	3–4 × 8–12	5–6/10	45 s	90 s	Introduction of plyometric tasks and alternating accelerations
Final	11–14	Transfer and application to gameplay	Mini-games with the ball, decision-making sprints, opposition challenges, mixed power, and agility circuits	3 × 8–10	6–7/10	30–45 s	90 s	Shortened rest to simulate intermittent effort, emphasis on open tasks, and soccer transfer

Notes. RPE = Rating of Perceived Exertion. All exercises were adapted to the age and skill level of the participants. Rest periods refer to the time allowed between stations and between circuit blocks.

**Table 2:** Descriptive statistics (Median and Interquartile Range) and baseline comparison between groups (Mann–Whitney U test).

Variable	Exp Median (IQR)	Ctrl Median (IQR)	*U*	*p*
Weight (kg)	31.00 (27.50–36.35)	28.40 (27.77–29.98)	70.5	0.354
Stature (cm)	135.00 (129.50–139.00)	139.50 (134.25–140.00)	63	0.193
BMI (kg/m^2^)	17.51 (16.23–19.34)	15.20 (14.63–16.25)	49	0.047
Handgrip R (kg)	12.25 (11.65–13.95)	12.17 (9.88–13.96)	76.5	0.526
Handgrip L (kg)	12.20 (10.70–13.10)	12.00 (9.20–15.11)	85	0.826
Illinois Agility (s)	18.24 (17.75–18.98)	17.63 (17.24–18.65)	65.5	0.236
Long Jump (cm)	131.50 (125.50–145.25)	147.25 (139.25–162.50)	49.5	0.051

Note. Values are presented as Median (Interquartile Range, IQR); BMI = Body Mass Index; Handgrip R/L = Right/Left handgrip strength; *U* = Mann–Whitney U test statistic; *p* = statistical significance.

**Table 3:** Within- and between-group changes for all outcomes (medians, IQR, changes), and net differences.

Variable	Group	T1: Pre Median (IQR)/Δ Pre–Mid	T2: Mid Median (IQR)/Δ Mid–Post	T3: Post Median (IQR)/Δ Pre–Post	% Pre–Post	*W/U*	*p*	*r*
BMI (kg/m^2^)	Exp	17.51 (16.23–19.34) /—	—/—	15.91 (15.12—17.99) /−1.59	−9.1%	*W =* 350.0	<0.001	0.85
	Ctrl	15.20 (14.63–16.25) /—	—/—	15.22 (13.96–15.80) /0.03	0.20%	*W =* 66.0	0.034	0.69
	Exp vs. Ctrl			ΔΔ = −1.29		*U =* 62.0	0.183	0.31
Handgrip R (kg)	Exp	12.25 (11.65–14.88) /+1.05	13.30 (12.25–16.30) /+3.25	16.55 (15.00–17.73) /+4.30	35.10%	*W =* 21.0	<0.001	0.89
	Ctrl	12.17 (9.88–13.96) /+1.03	13.20 (11.33–14.32) /+0.30	13.56 (11.44–16.51) /+1.39	11.40%	*W =* 18.0	0.11	0.54
	Exp vs. Ctrl			ΔΔ = +2.91		*U =* 37.5	0.011	0.58
Handgrip L (kg)	Exp	12.20 (10.70–13.10) /+1.23	13.08 (11.28–17.00) /+2.72	15.80 (14.93–17.03) /+3.60	29.50%	*W =* 28.0	<0.001	0.85
	Ctrl	12.00 (8.08–15.11) /+0.05	12.05 (10.80–15.30) /+0.63	12.68 (10.62–14.52) /+0.68	5.70%	*W =* 26.0	0.339	0.33
	Exp vs. Ctrl			ΔΔ = +2.92		*U =* 28.0	0.003	0.69
Illinois (s)	Exp	18.24 (17.75–18.99) /−0.05	18.19 (16.66–18.79) /−0.75	17.44 (16.46–17.99) /−0.80	−4.4%	*W =* 375.0	<0.001	0.98
	Ctrl	17.63 (17.24–18.65) /−0.07	17.56 (17.22–18.58) /−0.11	17.45 (16.94–18.26) /−0.21	−1.2%	*W =* 75.0	0.002	0.92
	Exp vs. Ctrl			ΔΔ = −0.59		*U =* 76.0	0.51	0.16
Long Jump (cm)	Exp	131.5 (125.5–145.3) /+2.5	138.5 (127.8–148.8) /+9.0	143.0 (133.5–155.0) /+11.5	8.70%	*W =* 48.0	<0.001	0.73
	Ctrl	147.3 (139.3–162.5) /−1.25	146.0 (136.0–162.0) /+1.75	147.75 (137.5–162.63) /+0.5	0.30%	*W =* 31.0	0.894	0.06
	Exp vs. Ctrl			ΔΔ = +11.0		*U =* 71.5	0.379	0.21

Note. All values are median (interquartile range, IQR) unless otherwise specified. Δ Pre–Mid = Absolute change in median from pretest (T1) to mid-intervention (T2); Δ Mid–Post = Absolute change in median from mid-intervention (T2) to posttest (T3); Δ Pre–Post = Absolute change in median from pretest (T1) to posttest (T3); % Pre–Post = Percent change in median from pretest to posttest: (Δ Pre–Post/Pre Median) × 100; *W =* Wilcoxon W statistic; *U =* Mann–Whitney U statistic; *p* = statistical significance; *r* = effect size r for the between-group comparison of change (ΔΔ, pre–post).

## Data Availability

The data presented in this study are openly available OSF in the Open Science Framework (OSF) at https://doi.org/10.17605/OSF.IO/XFR27.

## Abbreviations

The following abbreviations are used in this manuscript:

BMI	Body Mass Index
RAE	Relative Age Effect
IQR	Interquartile Range
HG-R	Right Handgrip Strength
HG-L	Left Handgrip Strength
SD	Standard Deviation
Δ	Change (difference between two time points)
*n*	Sample Size
